# Optimal Multi-Type Sensor Placement for Structural Identification by Static-Load Testing

**DOI:** 10.3390/s17122904

**Published:** 2017-12-14

**Authors:** Numa Joy Bertola, Maria Papadopoulou, Didier Vernay, Ian F. C. Smith

**Affiliations:** 1 ETH Zurich, Future Cities Laboratory, Singapore-ETH Centre, 1 CREATE Way, CREATE Tower, Singapore 138602, Singapore; papmara@gmail.com (M.P.); didiervrn@gmail.com (D.V.); 2 Applied Computing and Mechanics Laboratory (IMAC), School of Architecture, Civil and Environmental Engineering (ENAC), Swiss Federal Institute of Technology (EPFL), CH-1015 Lausanne, Switzerland; ian.smith@epfl.ch

**Keywords:** structural identification, measurement systems, sensors, model falsification, joint entropy, uncertainties, load tests

## Abstract

Assessing ageing infrastructure is a critical challenge for civil engineers due to the difficulty in the estimation and integration of uncertainties in structural models. Field measurements are increasingly used to improve knowledge of the real behavior of a structure; this activity is called structural identification. Error-domain model falsification (EDMF) is an easy-to-use model-based structural-identification methodology which robustly accommodates systematic uncertainties originating from sources such as boundary conditions, numerical modelling and model fidelity, as well as aleatory uncertainties from sources such as measurement error and material parameter-value estimations. In most practical applications of structural identification, sensors are placed using engineering judgment and experience. However, since sensor placement is fundamental to the success of structural identification, a more rational and systematic method is justified. This study presents a measurement system design methodology to identify the best sensor locations and sensor types using information from static-load tests. More specifically, three static-load tests were studied for the sensor system design using three types of sensors for a performance evaluation of a full-scale bridge in Singapore. Several sensor placement strategies are compared using joint entropy as an information-gain metric. A modified version of the hierarchical algorithm for sensor placement is proposed to take into account mutual information between load tests. It is shown that a carefully-configured measurement strategy that includes multiple sensor types and several load tests maximizes information gain.

## 1. Introduction

While infrastructure is ageing, available economic and environmental resources are decreasing. Therefore, an optimal infrastructure management strategy is needed. Due to the justifiably conservative nature of design and construction of large civil structures, most structures have a significant amount of reserve capacity. Unfortunately, this reserve is largely unquantified, resulting in sub-optimal asset-management decisions. For example, knowledge of load capacity of bridges can be exploited to extend lifetimes of existing structures, optimize retrofit designs and prioritize inspection and maintenance activities.

Field-measurements, collected during load testing and through ambient vibration monitoring, have been extensively used in the last decades to identify bridge characteristics [1]. Interpretation of the data provided by sensors is critical to identify accurate structural models and subsequently, to estimate bridge reserve capacity. Such interpretation is a type of inverse engineering where causes (behavior models and their inputs) are determined from effects (measurements). This type of inference is fundamentally ambiguous; many causes may explain the same effect, especially when modelling uncertainties are important at sensor locations. The difficulties associated with the inverse problem of structural identification have been recognized by [2,3] amongst many others.

The aim of model-based structural identification is to use field measurements to improve knowledge of the real behavior of structures. Several data-interpretation techniques exist to perform this task, such as residual minimization [4] and Bayesian updating [5,6]. Such traditional model calibration methodologies cannot be justified for large civil structures [7]. They often result in biased identification due to important systematic uncertainties that modify correlation values between measurement points. To overcome challenges associated with inverse problems, a multi-model approach was proposed by [8,9]. In this method, model-updating results consist of a set of candidate models that explain the measurements taken from a structure.

A probabilistic extension, called error-domain model falsification (EDMF) was presented by [10]. In this methodology, a population of model instances are generated according to prior knowledge and engineering judgement. Threshold bounds are determined probabilistically using the Monte Carlo method and a target confidence level. Then, they are used to falsify model instances that significantly differ from measured values. Systematic uncertainties are transparently included and the use of uniform probability distributions increases robustness to unknown uncertainty correlations [10]. This methodology has been successfully applied to other fields such as wind simulation around buildings [11], leak detection in water-supply networks [12] and performance following earthquake damage [13].

Model updating outcomes depend on the choice of sensor types and locations. However, most of the practical applications of structural identification involve placement of sensors based on engineering judgement and experience. More rational studies on optimal sensor locations for structural identification have been carried out using information theory to improve model-parameter estimation. Various approaches have been used: maximizing the determinant of Fisher information matrix [14,15] and either minimizing the information entropy in posterior model-parameter distribution [16,17] or maximizing information entropy in multiple-model predictions [18,19]. Although entropy-based approaches have shown to be powerful to find the optimal sensor configuration, few studies have included systematic modelling uncertainties and information that is shared amongst sensors.

The effect of spatially-correlated prediction errors were included by [20] to correct the information entropy of model-parameter posterior distribution, which was used as the objective function in the sensor placement methodology. The authors have shown that the minimum distance between sensors is controlled by the spatial correlation length of the prediction errors. By accounting for it, the redundancy of information of neighboring sensors can be avoided. In addition, they observed that an assumption of uncorrelated prediction errors in models may lead to sub-optimal sensor configurations. Limitations associated with potential redundancy of information using individual-sensor entropy metric was underlined by [21]. The importance of the mutual information between sensors in optimal configuration of multi-type of sensors was shown by [22].

Another approach, presented by [23] and extended by [24], used simulated measurements to provide probabilistic estimations of the expected number of candidate models obtained with a sensor configuration. The aim was to find the sensor configuration that minimizes the expected number of candidate models. Simulated measurements are generated based on the model instances adding a random value taken from the combined uncertainties. Sensor locations were evaluated using respectively 95% and 50% quantiles of the expected candidate-model-set size. However, the procedure is computationally costly [25], because it requires the execution of the falsification procedure for a large number of simulated measurements and sensor locations.

The problem of finding the optimal sensor configuration is usually formulated as a discrete problem. As the number of possible sensor configurations is very large, an exhaustive search for the best configuration is exponentially complex with respect to the number of sensors. Some studies proposed global-search optimization algorithms to determine optimal solutions [19]. However, most authors preferred to reduce the computational effort using greedy optimization algorithms [26]. 

Entropy calculations based on sequential optimization strategies have involved inefficient search methods, assumed constant uncertainty levels at all sensor locations and most researchers have disregarded the mutual information between sensor locations. A methodology involving a hierarchical algorithm to examine placement alternatives efficiently and incorporating spatial distributions of modelling uncertainties was introduced by [21]. The authors also proposed to maximize the joint entropy between sensor locations to account for mutual information. This methodology was able to improve model predictions at unmeasured locations compared with a methodology based on individual entropy maximization. This sensor placement algorithm was successfully applied to sensors for wind-around-building predictions [27]. Sensor configurations using a multi-criteria decision-making approach and various information-gain metrics was evaluated by [28], including the prediction range and type I and type II errors. 

As highlighted by [20], the optimal sensor placement for model-parameter estimation depends on the loading. Previous work has focused only on the information gained by adding a sensor to the sensor configuration and not by performing additional load tests using the same sensor configuration. In work on model falsification, the next sensor to add in the sensor configuration was associated with a pre-defined load test [23,24]. In these studies of sensor placement for structural identification, mutual information between multiple load tests is not considered within the sensor placement methodology.

This study presents a measurement-system design methodology to identify the best sensor locations and sensor types using information from several static load tests. First, the EDMF methodology for structural identification is presented. Then, the hierarchical strategy for sensor placement is adapted and extensions to the sensor placement algorithm are proposed. Optimal sensor configurations for independent static-load tests are computed. Then, two modifications of the sensor placement algorithm which take into account information for multiple static load tests are proposed. Finally, sensor placement strategies are illustrated and evaluated on a full-scale bridge.

## 2. Materials and Methods

### 2.1. Background—Error-Domain Model Falsfication

Presented by [10], error-domain model falsification (EDMF) is a structural identification methodology. Within model parameter sets, multiple model instances are generated and falsified if their predictions differ significantly from field measurements. First, an initial-model instance population is generated from engineering judgment and prior knowledge. Model instances are instantiations of a model class, in which several combinations of primary parameter values $\theta_{k}{\;=\;[\theta }_{1}{,\theta }_{2}{,\text{…},\theta }_{n}{]}^{T}$ are assigned in order to generate an initial set of model instances Ω. Then, model instance predictions are compared with field measurements of the structural response in order to identify candidate models among the initial set of the model instance population. Modelling and measurement uncertainties are combined to determine threshold boundaries [9]. Threshold boundaries are defined using the combined distribution of uncertainties and a target reliability of identification. Model instances are falsified if the residual value between predictions and measurements exceeds the boundaries at one or more sensor locations. 

For each measurement location, $i\in \left\{1,\ldots ,n_{y}\right\}$, model predictions and measurements are linked to the true behavior using Equation (1). $R_{i}$ corresponds to the real responses of a structure (unknown) and ${\hat{y}}_{i}$ to the measured value at location i. Using finite element analysis (FEA), predictions $g_{k}\left(i,\Theta_{k}\right)$ of the model class G_k_ is evaluated at location i. $\Theta_{k}$ is the set of instances of the parameter vector $\theta_{k}$, $U_{i,g_{k}}$ and $U_{i,\hat{y}}$ correspond to model-prediction uncertainties and measurement uncertainties, respectively:

(1) $$g_{k}\left(i,\Theta_{k}\right)\;+\;U_{i,g_{k}}=\;R_{i}=\;{\hat{y}}_{i}+\;U_{i,\hat{y}}\;\forall i\in \;\left\{1,\ldots ,n_{y}\right\}$$

Equation (1) may be rearranged to Equation (2), where $U_{i,c}$ is the difference between the modelling and measurement uncertainties. The left-hand side of Equation (2) represents the difference between a model prediction and a measurement:

(2) $$g_{k}\left(i,\Theta_{k}\right)-{\hat{y}}_{i}=\;U_{i,c}=\;U_{i,\hat{y}}-\;U_{i,g_{k}}$$

The selection of candidate models representing realistic sets of model-parameter values, involves falsifying all model instances for which predictions cannot explain measurement data, given combined uncertainties and a target reliability of identification ϕ. The set of candidate models obtained after falsification is defined using Equation (3), where $\Omega_{k}^{\prime \prime }$ is the candidate model set (CMS) made up of initial model instances, which have not been falsified at one or more measurement locations. [$u_{i,\mathrm{low}}$, $u_{i,\mathrm{high}}$] are the upper and lower threshold bounds. They represent the shortest intervals, including a probability of identification $\phi^{1/n_{y}}$, through the probability density function (PDF) of combined uncertainties $f_{U_{i}}\left(u_{i}\right)$ at each measurement location:

(3) $$\Omega_{k}^{\prime \prime }=\left\{\theta_{k}\in \Omega_{k}|\forall i=\;1,\ldots ,n_{y}\;u_{i,low}\leq g_{k}\left(i,\Theta_{k}\right)-{\hat{y}}_{i}\leq u_{i,high}\right\}$$

The Šidák correction [29] is used to maintain a constant level of confidence when multiple sensor measurements are compared with model instance predictions (Equation (4)):

(4) $$\forall i=\;1,\ldots ,n_{y}:\phi^{1/n_{y}}=\int_{u_{i,low}}^{u_{i,high}}f_{U_{i}}\left(u_{i}\right)du_{i}\;\forall i\in \left\{1,\ldots ,n_{y}\right\}$$

All model instances that belong to the CMS, $\Theta_{k}\in \Omega_{k}^{\prime \prime }$, are labeled as candidate models. Since so little information is usually available to describe the form of modelling-uncertainty distributions, every candidate model is equally likely to be the correct model [30]. Thus, they are assigned an equal probability as expressed in Equation (5):

(5) $$\mathrm{Pr}\left(\Theta_{k}\in \Omega_{k}^{\prime \prime }\right)=\frac{1}{\int^{\text{​}}\theta_{k}\in \Omega_{k}^{\prime \prime }\;d\theta_{k}}$$

Falsified model instances, which correspond to model instances that do not belong to the CMS, are assigned a null probability (Equation (6)):

(6) $$\mathrm{Pr}\left(\Theta_{k}\notin \Omega_{k}^{\prime \prime }\right)=0$$

Consequently, $\Theta_{k}^{\prime \prime }$ is the set of random variables describing the parameter values of the candidate model instances given measurement data. Its PDF is defined using Equation (7):

(7) $$f_{\Theta_{k}^{\prime \prime }}=\{\begin{array}{c} \frac{1}{\int^{\text{​}}\theta_{k}\;\in \;\Omega_{k}^{\prime \prime }d\theta_{k}},\;if\;\Theta_{k}\in \Omega_{k}^{\prime \prime }\\ 0,\;otherwise \end{array}$$

If all initial model instances generated are falsified, the entire model class is falsified, then $\Omega_{k}^{\prime \prime }=\;\emptyset$. Thus, no models are compatible with observations given model and measurement uncertainties. Possible reasons are an incorrect model-class definition, incorrect uncertainty estimates, or wrong initial parameter values [31]. This particular case highlights one of the main advantages of EDMF compared with traditional structural-identification approaches. In this situation, the results of EDMF leads to a re-evaluation of starting assumptions and, often, a new model class is generated.

Concerning the identifiability of the methodology, a key feature of EMDF is the following: if only a subset of sensors is considered and a small influence of the Šidák correction on thresholds is assumed, the candidate-model-set (CMS) value range will be wider than using all sensors due to the smaller decrease of parameter bounds compared with using all sensors. The resultant CMS using all sensors will still be part of the larger CMS obtained with a subset of sensors, which will lead to conservative conclusions in terms of type-I error (falsely rejecting a candidate model).

### 2.2. Sensor Placement Strategy

Assessing a full-scale infrastructure, such as estimating the reserve capacity of a bridge, requires the estimation of various unknown physical properties and boundary conditions. The aim of field measurements is to enhance knowledge of model parameters and improve structural assessments. The choice of sensor locations is fundamental for structural identification. A sensor placement strategy is usually developed to identify optimal sensor configurations prior to measuring when limited knowledge of the model parameter values is available.

A model-based sensor placement strategy requires several steps. First, a numerical model, such as a finite element model of a bridge, is built to obtain quantitative predictions of measurable variables, such as deflection, strain, or inclination at each possible sensor location. As the numerical model always requires geometrical and mathematical simplifications, a significant degree of non-parametric uncertainty is involved, which needs to be evaluated. Then, sensitivity analysis is employed to evaluate the effects of variation in model-parameter values on model predictions. A small number of parameters, which have the highest impact on predictions, are then selected. Several possible load tests are designed and multiple model instances are generated using a sampling technique to obtain a discrete population of possible model-parameter values within plausible ranges. For each load test, model instance predictions are computed and each instance is part of the initial model set. The initial model set is the dataset used in the sensor placement strategy.

In this section, several sensor placement strategies are presented. First, two objective functions for sensor placement—single-sensor information entropy and joint entropy—are presented. Then, originally developed for the prediction of wind around buildings, the initial version of the hierarchical algorithm is introduced. As this sensor placement algorithm was designed for wind assessment, two modifications are proposed for taking into account information from several static load tests.

#### 2.2.1. Sensor Placement Objective Function

##### Information Entropy

The information obtained from prediction data is a major criterion for evaluating possible sensor locations. This can be evaluated using entropy from information theory (also known as Shannon’s entropy or information entropy). The information entropy $H\left(y_{i}\right)$ is a measure of disorder in information content (Equation (8)), where $y_{i}$ is an output variable, such as the deflection at a sensor location i, $P\left(y_{i,j}\right)$ is the probability of the jth interval of a variable’s distribution with $j\in \left\{1,\ldots ,N_{I,i}\right\}$, and $N_{I,i}$ is the maximum number of intervals at the location i:

(8) $$H\left(y_{i}\right)=-\sum_{j=1}^{N_{I,i}}P\left(y_{i,j}\right)\log_{2}P\left(y_{i,j}\right)$$

**Figure 1:**
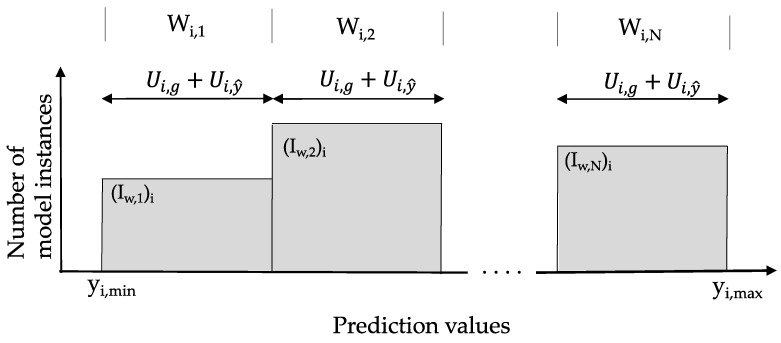
Construction of model instance subsets within an interval width W at the measurement location i using modeling ($U_{i,g}$) and measurement ($U_{i,\hat{y}}$) uncertainties.

At a possible sensor location, the evaluation of the information entropy requires creation of subsets of model instance predictions. The construction of subsets of model instances at a location i is presented in Figure 1 with the number of model instances as the vertical axis and the value of predictions as the horizontal axis. N intervals (I_w,i_) are generated between the minimum and the maximum prediction of model instances at a possible sensor location. W_i,j_ represents the width of the interval and is equal to the sum of measurement ($U_{i,\hat{y}}$) and modelling ($U_{i,g}$) uncertainties (at 95%) at this location. The width of the intervals is constant for a sensor location i.

At each sensor location, the information entropy is computed from Equation (8) through first counting the number of model instances m_i,j_ in a subset, for which predictions fall within each interval and then calculating the probability of the interval as $P\left(y_{i,j}\right)=m_{i,j}/\sum^{\text{​}}m_{i,j}$. A location with a high information entropy value of model predictions is considered as a good location [18]. The uniform distribution is the maximum entropy distribution on a given range of model instance prediction values.

Model instances in a subset cannot be distinguished from each other using an in-situ measurement falling in the middle of the interval during the falsification process. Thus, model instances in the same subset might not be discriminated using this sensor location. Another location is needed to further subdivide these subsets.

##### Joint Entropy

The joint entropy is a more recent sensor placement objective function that has been proposed for system identification by [21]. The joint entropy is an information entropy measure associated with a set of locations, while assessing the mutual information of the locations. For a set of two sensors, it is defined in Equation (9), where $k\in \left\{1,\ldots ,N_{I,i+1}\right\}$ and N_I,i+1_ is the maximum number of intervals at the i + 1 location and $i+1\in \left\{1,\ldots ,n_{s}\right\}$ with $n_{s}$ the number of potential sensor locations:

(9) $$H\left(y_{i,i+1}\right)=-\sum_{k=1}^{N_{I,i+1}}\sum_{j=1}^{N_{I,i}}P\left(y_{i,j},y_{i+1,k}\right)\log_{2}P\left(y_{i,j},y_{i+1,k}\right)$$

The joint entropy is less than or equal to the sum of the individual entropies of the locations in the set (Equation (10)), where I is the mutual information between sensor i and i + 1:

(10) $$H\left(y_{i,i+1}\right)=H\left(y_{i}\right)+H\left(y_{i+1}\right)-I\left(y_{i,i+1}\right)$$

#### 2.2.2. Hierarchical Algorithm

A hierarchical algorithm for sensor placement using the concept of joint entropy was introduced in [21]. The hierarchical algorithm is a sequential algorithm (greedy search) in which model instances are organized in a tree structure. At the root is the initial model set, and branches contain subsets of model instance predictions. Branches from a node represent separations of the parent model set into smaller subsets that can potentially be divided using measurements from the new sensor added to the configuration. This allows calculations of joint entropy of sensor configurations while avoiding exponential complexity, reducing the computational effort.

**Figure 2:**
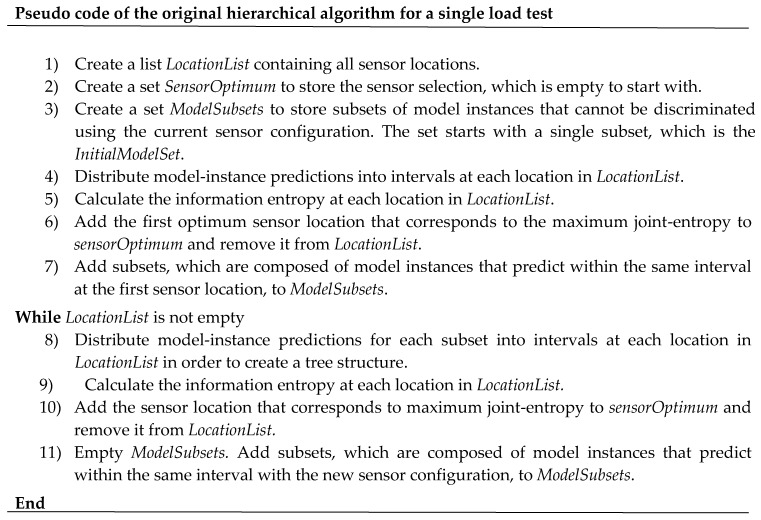
Pseudo-code for the original hierarchical algorithm for a single load test (adapted from [21]).

The pseudo-code of the original hierarchical algorithm is presented in Figure 2. This algorithm can accommodate a single load test only. The first sensor (i = 1) is selected with the information entropy objective function. However, for i > 1, the location with the maximum joint entropy of the configuration is selected. This sensor placement algorithm takes into account mutual information between sensors because of the joint entropy objective function. It was shown to perform better than traditional sequential algorithms with forward or backward strategies [21].

**Figure 3:**
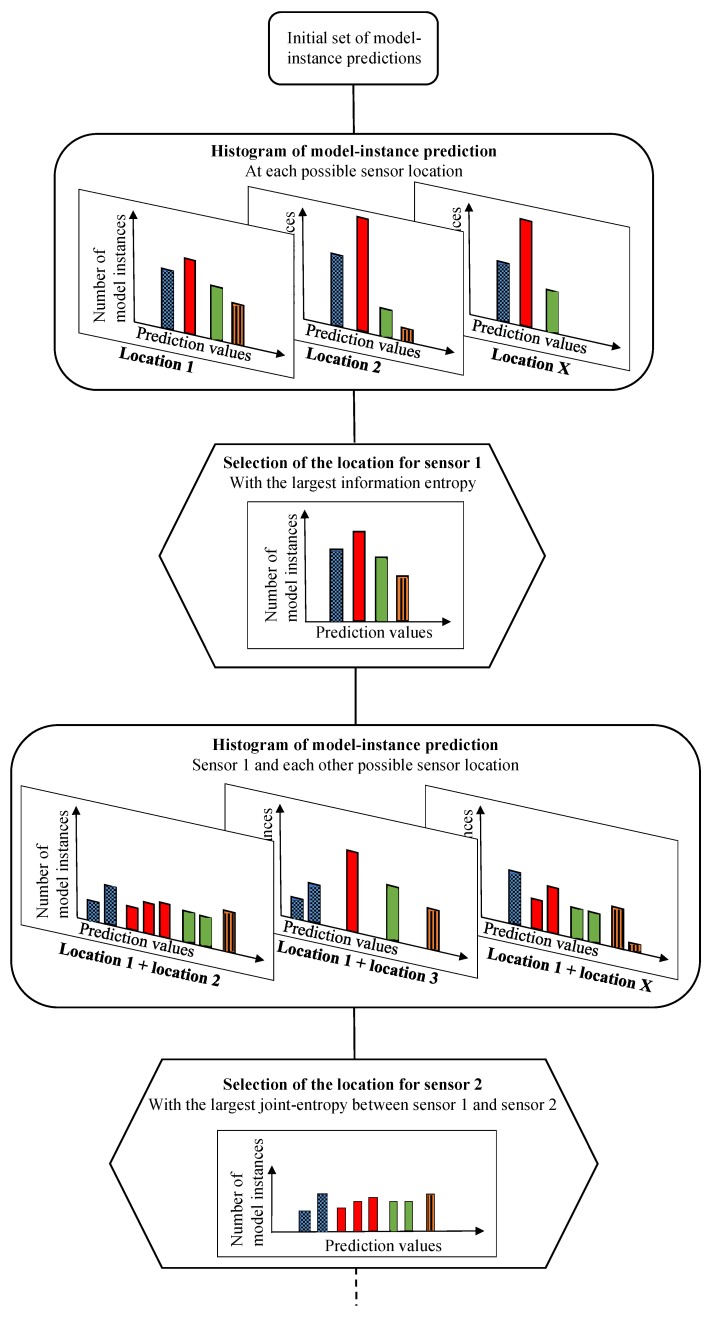
Schematic of the hierarchical algorithm for sensor placement where histograms at possible sensor locations are composed of subset of model instances depicted in distinctive bars.

A schematic of the tree structure of the hierarchical algorithm is presented in Figure 3. At the top of the figure is the initial set of model instance predictions at possible sensor locations. At each possible sensor location, a histogram of predictions of model instances in the initial set is generated, following Figure 1. Histograms are composed of subset of model instances depicted in distinctive bars. In Figure 3, clear spaces between the distinctive bars are added for clarity only. In most practical cases, model prediction values are continuous and, thus, the subset bars of the histograms would be touching.

Once histograms of model predictions are generated at each possible location, the location with the largest information entropy value is selected and Sensor 1 is added to the sensor configuration. To select the second sensor, information from the remaining sensors is used to further divide each subset of model instances of Sensor 1. The configuration of Sensor 1 and Sensor 2 with the largest joint entropy is selected and Sensor 2 is added to the sensor configuration. The process is repeated until all possible sensor locations are selected.

This process is repeated at every iteration by adding a sensor to the sensor configuration, forming a hierarchy of model subsets. At each stage of the sensor placement, a location is added to the configuration sensor optimum that has the highest potential in dividing the existing subsets of model instances into smaller subsets. The maximum number of iterations required is independent of the number of combinations of sensor locations and is equal to the number of possible subdivisions; the upper bound of this quantity is equal to the maximum number of model instances among all subsets of Sensor 1.

#### 2.2.3. Modification of Hierarchical Algorithm for Multiple Load Tests

The original version of the hierarchical algorithm (Figure 2 and Figure 3) accommodates only a single load test. To consider information from various load tests, two modifications of the algorithm are presented in this section. The first modification includes only minor changes in the code to enable the hierarchical algorithm to select best sensor locations considering only the load test that maximizes their information content. The second modification includes significant changes in the code to enable the hierarchical algorithm to select the best sensor location considering all load tests.

##### First Modification—Minor Changes to the Original Hierarchical Algorithm

**Figure 4:**
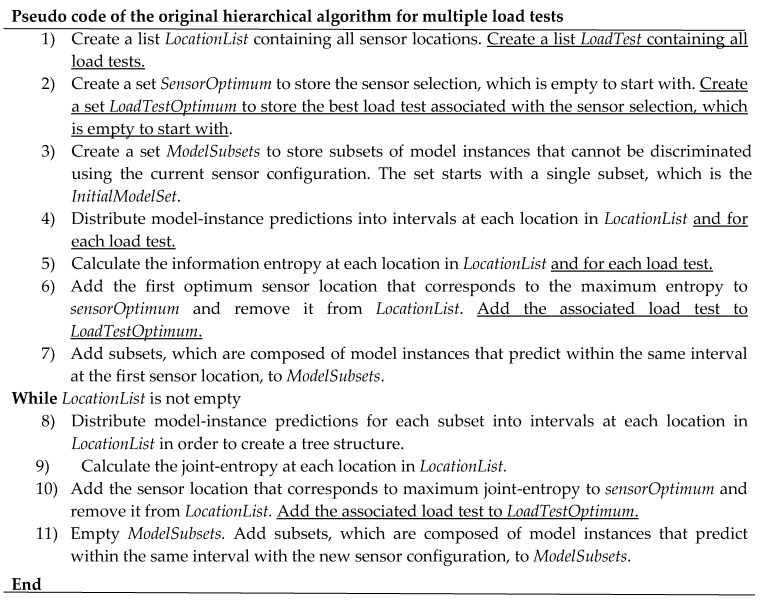
Pseudo-code of the original hierarchical algorithm for multiple load tests within the first modification.

The first modification enables the algorithm to select best sensor locations considering only the load test that maximizes their information content. The pseudo-code for the hierarchical algorithm for multiple load tests is presented in Figure 4. Differences with the pseudo-code of the hierarchical algorithm for a single load test (Figure 2) are underlined. The tree structure remains unchanged. The sensor selection is based on the joint entropy of a location and a load test. Therefore, it allows the algorithm to select the best load test among a set of various load tests for each sensor location. However, the algorithm does not consider information provided by the other load tests at one sensor location. This approach to consider multiple load tests was used by [23,24] using a backward sequential algorithm instead of the hierarchical algorithm. As this modification is minor, this version of the hierarchical algorithm is called original hierarchical algorithm for multiple load tests.

##### Second Modification—Major Changes to the Hierarchical Algorithm

The first modification of the algorithm selects the sensor location associated with the load test that maximizes its information content. However, this location may not perform well for other load tests and another location may perform better when considering information from all load tests. Therefore, a modification to the hierarchical algorithm considering information provided by all load tests is justified.

In the original hierarchical algorithm, the joint entropy is an information entropy measure associated with a set of locations, while assessing the mutual information between locations. With the version proposed in this study, it is possible to calculate the joint entropy at a single location when multiple load tests are considered, through assessing the mutual information between load tests. In the proposed modification of the hierarchical algorithm, the assessment of mutual information between sensors remains applicable if multiple sensor locations are considered.

For a sensor location and a set of two load tests, it is defined in Equation (11), where $j\in \left\{1,\ldots ,N_{I,i_{l}}\right\}$, and $N_{I,i_{l}}$ is the maximum number of intervals at the location i associate with a load test l, $k\in \left\{1,\ldots ,N_{I,i_{l+1}}\right\}$ and $N_{I,i_{l+1}}$ is the maximum number of intervals at the location i associated with another load test and $l+1\in \left\{1,\ldots ,n_{\mathrm{LT}}\right\}$ with $n_{\mathrm{LT}}$ the number of potential load tests:

(11) $$H\left(y_{i_{l},i_{l+1}}\right)=-\sum_{k=1}^{N_{I,i_{l+1}}}\sum_{j=1}^{N_{I,}i_{l}}P\left(y_{i_{l},j},y_{i_{l+1},k}\right)\log_{2}P\left(y_{i_{l},j},y_{i_{l+1},k}\right)$$

The joint entropy is less than or equal to the sum of the individual entropies of the sensor location with individual load test in the set, hence, Equation (10) is modified as follows:

(12) $$H\left(y_{i_{l},i_{l+1}}\right)=H\left(y_{i_{l}}\right)+H\left(y_{i_{l+1}}\right)-I\left(y_{i_{l},i_{l+1}}\right)$$

where I is the mutual information between a sensor associate with the load test l and the same sensor associated with the load test l + 1.

**Figure 5:**
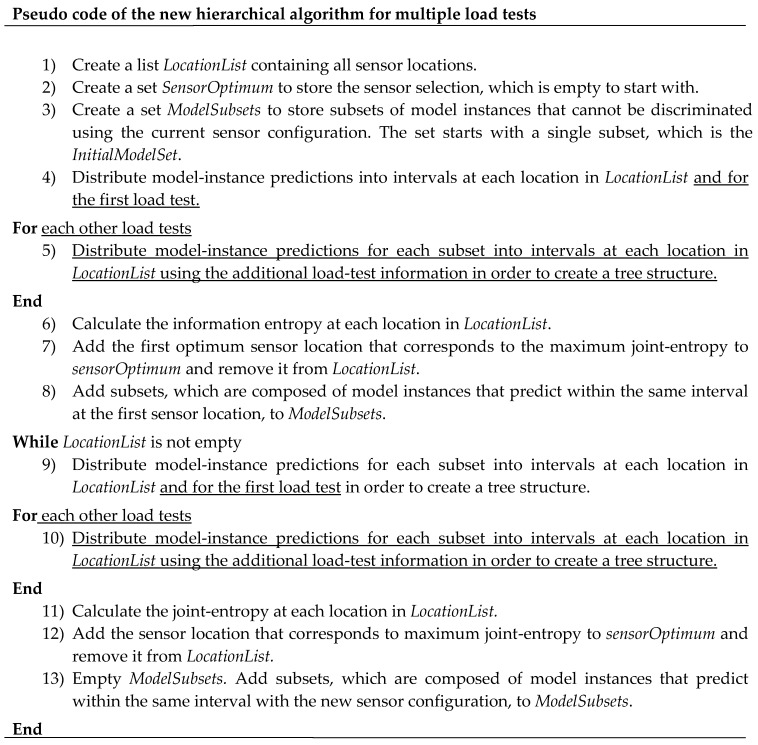
Pseudo-code of the new hierarchical algorithm for multiple load tests within the second modification.

The pseudo-code of the new hierarchical algorithm is presented in Figure 5. Differences with the pseudo-code of the hierarchical algorithm for a single load test (Figure 2) are underlined. A new loop is added to distribute model instance predictions into sub-intervals defined using the additional load tests. In the fifth point, the algorithm distributes previous subsets of model instances, obtained with the first load test, using model instance predictions obtained with a new load test. Consequently, the algorithm evaluates the mutual information between load tests at the same sensor location. As this modification includes major changes to the original hierarchical algorithm, this sensor placement algorithm is called the new hierarchical algorithm for multiple load tests.

## 3. Results

### 3.1. Case Study

**Figure 6:**
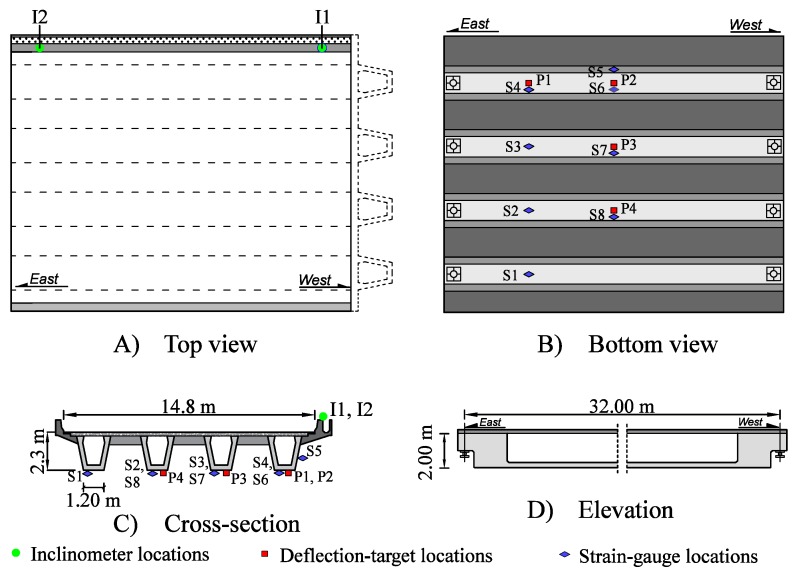
Bridge geometry showing the sensor configuration; (**A**) Top view; (**B**) Bottom view; (**C**) Cross-section; (**D**) Elevation.

A full-scale case study was performed on a 32-year-old bridge in Singapore. The principal characteristics of the bridge are presented in Figure 6. The pre-stressed concrete bridge is composed of four beams carrying three unidirectional traffic lanes over a simply-supported span of 32 m.

The sensor configuration consisted of two inclinometers (I_i_) on the parapet, four deflection targets (P_i_), and eight strain gauges (S_i_) on the main girders (Figure 6A,B). A laser tracker was positioned on the road below the bridge and used to measure deflections. Seven of the eight strain gauges were installed on the bottom face of the girder in the direction of the principal stress at mid-span or quarter span of the bridge. The aim was to measure the stress in the main girders. The last strain gauge (S6), was installed horizontally on the web, close to the expected neutral-axis location, at mid-span of the most loaded girder. The aim was to define the location of the neutral axis. The sensor configuration was chosen based on engineering judgment and practical considerations. In total, fourteen possible sensor locations were investigated for three types of sensors.

Five primary parameters were identified as having the most influence on measurements: the Young’s modulus of site-cast concrete of the deck E_con_, the Young’s modulus of the precast concrete of the beams E_pre_, the Young’s modulus of the concrete of the barrier E_bar_, the rotational stiffness of the bearing devices K_rot_, and the vertical stiffness of the bearing devices K_lon_. Their plausible ranges of values are estimated using engineering heuristics and are presented in Table 1. To reduce model simplification uncertainties, non-structural elements, such as the asphalt pavement, are included in the finite-element solver. 1000 initial model instances were generated using Latin Hypercube sampling (LHS) within this five-parameter space.

Upper and lower bounds of model-class uncertainties and measurement uncertainties are presented in Table 2. All uncertainties are considered with a uniform distribution and are estimated based on engineering judgment, sensor-supplier information, and heuristics. The Monte-Carlo (MC) approach is used to combine modelling and measurements to a single combined-uncertainty distribution, through randomly selecting a value from each distribution. This process is usually repeated 1,000,000 times to generate the combined distribution. The number of repetitions is fixed so that thresholds do not change significantly if this number is increased.

**Figure 7:**
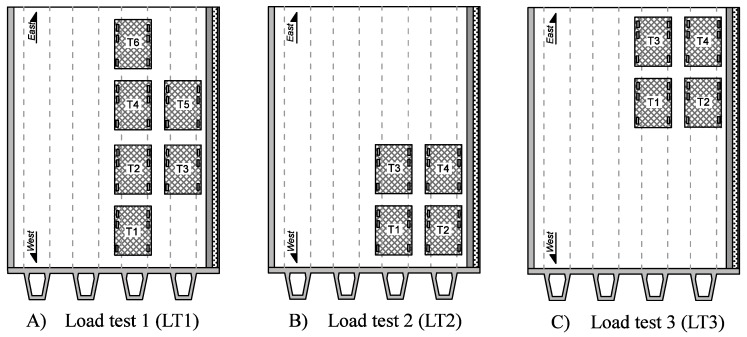
Load test presentation; (**A**) first load test; (**B**) second load test; (**C**) third load test.

Three static load tests were performed on the bridge and the truck configurations are presented in Figure 7. Load tests are composed of trucks of approximately 32 tons within three axles. The repartition of the load is 1/5; 2/5; 2/5 starting from the front axle. The first load test (LT1) is composed of six trucks symmetrically disposed on the bridge. The aim is to maximize the deflection and strain in the precast beams. The second and third load tests (respectively, LT2 and LT3) are non-symmetrical load tests, where four trucks are successively disposed only on one side of the bridge. The aim of these load tests is to maximize the inclination at the bridge supports.

### 3.2. Load Test Comparison in Terms of Information Gain

In this section, each load test is considered independently. First the information entropy of each sensor is independently compared for each load test. Then, the sensor ranking from the original hierarchical algorithm is compared in terms of the sensor configuration and joint entropy values.

#### 3.2.1. Information Entropy of Sensors

**Figure 8:**
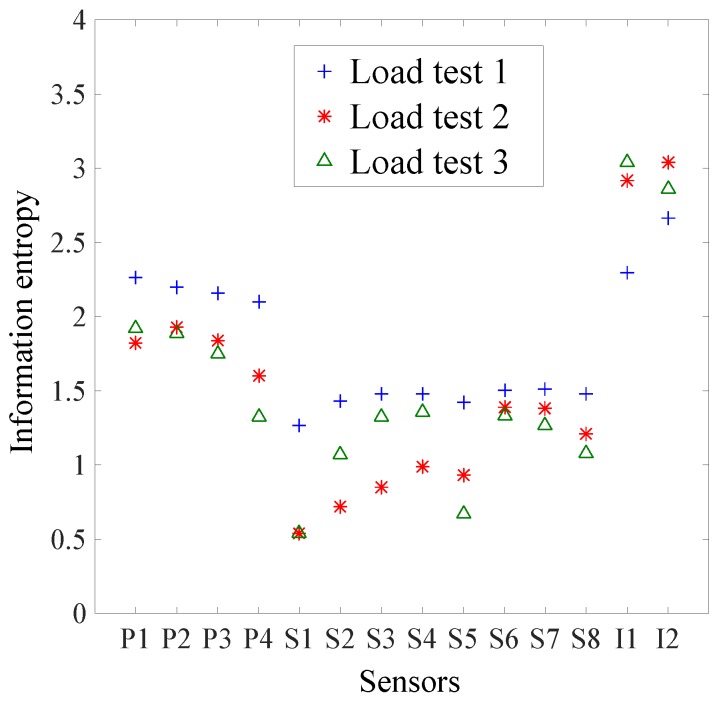
Sensor information entropy at each sensor location for each load test.

For each load test, the information entropy at each sensor location is presented in Figure 8. The vertical axis presents the information entropy value and the horizontal axis presents the sensor locations shown on Figure 6. To compute the information entropy, two components are the determining factors. Firstly, the distribution of model instance predictions influences the information entropy value. This distribution mostly depends on the sensor location, bridge characteristics, and the choice of primary parameters for structural identification. Secondly, the definition of measurement and modeling uncertainties influencing the width of intervals, and thus the value of information entropy.

Globally, the inclinometers have the largest information entropy, regardless of the load test. Then, the deflection targets have larger entropy values than strain gauges. As each type of sensor has specific characteristics, the modeling and measurement uncertainties differ (Table 2) and explain the difference of information entropy values between sensor types. The inclinometer was proportionally more precise (sensor precision) than the strain gauge and the deflection target, which explains the highest information entropy of inclinometers compared to other sensor types. Strain gauges have larger uncertainty values associated with sensor orientation, sensor installation, and spatial variability compared to the deflection target and, thus, have smaller information entropy values.

Concerning the deflection target, the load test 1 (LT1) has larger information entropy for all sensor locations than load test 2 (LT2) and load test 3 (LT3). LT2 and LT3 have very similar results. For the strain gauges, the LT1 has slightly larger information entropy for all sensor locations than LT2 and LT3. LT3 has slightly smaller entropy than LT1 for the strain gauges located just below the trucks (S2 to S4). Regarding the inclinometers, LT2 and LT3 have the largest information entropy for inclinometers I1 and I2, respectively. As LT1 has smaller entropy than LT2 and LT3, it shows that the inclinometers are more powerful using a load test with trucks close to the support than at midspan, even if a smaller number of trucks is involved. Except for the inclinometers, sensor locations of LT1 have larger information entropy due to the larger signal-to-noise ratio of this load test.

For LT1, the difference of information entropy between sensors of the same type at various locations is small. This shows that for a symmetrical load test, such as LT1, the information entropy of the sensor is more related to the sensor type than the sensor location, due to the difference in uncertainty values between sensor types.

In order to understand the factors influencing the difference of information entropy values, two locations will be explained as examples: P4 between LT1, LT2, and LT3 and S4, S5, S5 for LT3. Concerning P4, it is observed that the normalized spread of predictions—defined as the difference between the maximum and minimum of predictions divided by the average value of predictions—is much larger for LT1 compared to LT2 and LT3. This means that the 1000 model instances are distributed more uniformly over the intervals and, thus, the information entropy value is larger for LT1. Additionally, even if LT2 and LT3 have a similar truck disposition, the axle-load locations differ slightly and could explain the difference between LT2 and LT3 at P4 location. Concerning strain gauges for LT3, it is observed that predictions are much lower for S5 compared to S4 and S6. This is explained by the location of the sensor: horizontally oriented on the web close to the neutral axis of the girder instead of on the bottom face. As the sensor precision and sensor repeatability (Table 2) are defined in absolute values, the number of intervals for this specific location is smaller. Hence, the information entropy value is smaller compared with sensor locations S4 and S6.

#### 3.2.2. Sensor Ranking

**Figure 9:**
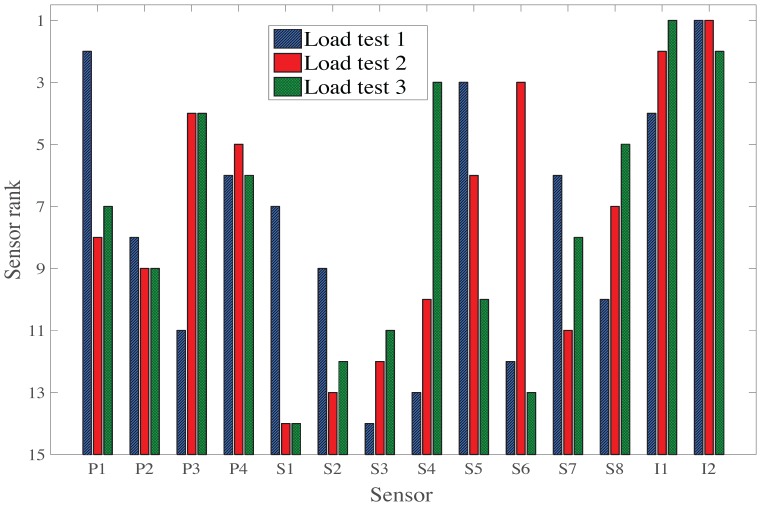
Sensor ranking for three load tests.

The selection of sensors is presented in Figure 9 as a histogram with the sensor rank in the vertical axis and the sensor identification in the horizontal axis. Results of each load test are presented with distinctive bars. Sensors were selected using the original hierarchical algorithm for the single load test (Figure 2).

The order of sensor selection, as well as the type of sensor, differs between the three load tests. For all load tests, an inclinometer is selected as the first sensor due to the large information entropy of this sensor type (Figure 8). Additionally, for all load tests, the hierarchical sensor placement algorithm selects sensors of three different types for the first four sensors, showing that each type of sensors provides unique information.

For LT2 and LT3, the inclinometers are selected for the first and second position. As the main difference between these load tests is the inverse position of the trucks on the bridge, this shows that the optimal sensor placement directly depends on the design of the load tests. Since the order of sensor selection differs between the load tests, a methodology for sensor placement taking into account multiple load tests is justified.

#### 3.2.3. Joint Entropy of Sensor Configurations

**Figure 10:**
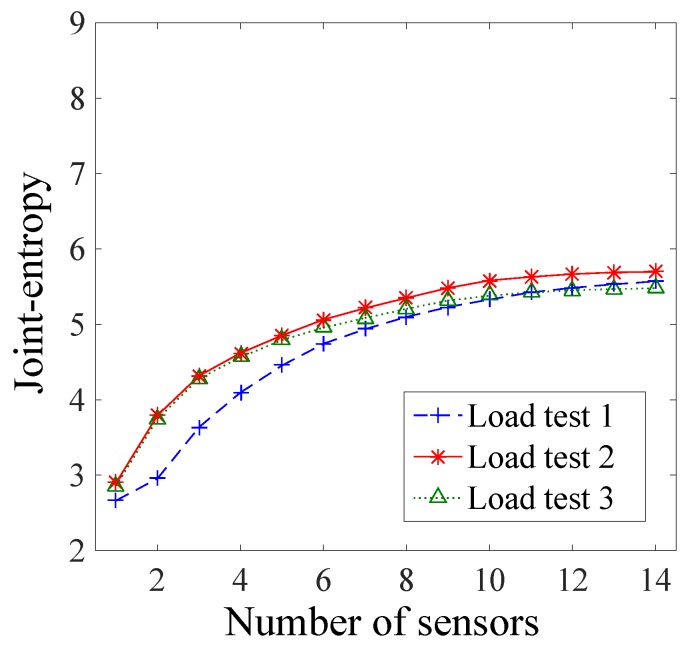
Joint entropy of the sensor configuration as a function of the number of sensors for each load test.

Sensor configurations from various load test configurations are compared using the joint entropy metric. The joint entropy is the objective function of the sensor placement algorithms used. It represents the ability of a sensor configuration to discriminate model instance predictions. The joint entropy of the sensor configuration for each load test is presented in Figure 10 as a function of the number of sensors.

LT2 has the largest joint entropy for any number of sensors in the sensor configuration, showing that LT2 is the most effective load test in terms of maximizing joint entropy. For a small number of sensors (N_s_ < 8), LT2 and LT3 present similar results and perform better than LT1, which has the smallest joint entropy value, because the inclinometers are more powerful in these load tests.

The joint entropy of LT3 differed from LT2, for the last eight sensors in the configuration, and the joint entropy values dropped below those of LT1 after the eleventh sensor. For a large number of sensors in the configuration (N_s_ > 10), the joint entropy is similar for all load tests. It is therefore concluded that for a large number of sensors the LT1 performs as well as the other load tests because the strain gauges and deflection targets are more effective (Figure 8). Thus, if only a single load test must be performed and a large number of sensors is available, the choice between LT1, LT2, and LT3 will not significantly influence the results in maximizing the joint entropy values. Nevertheless, even if the joint entropy estimates are similar, the information gained from each load test may differ.

### 3.3. Sensor placement-Strategy Implementation with Consideration of Information from Multiple Static Load Tests

In this section, optimal sensor placements from the two modified hierarchical algorithms for multiple load tests are presented. The sensor ranking of each algorithm is presented and sensor configurations are compared using joint entropy as a metric of information gain. 

#### 3.3.1. Original Hierarchical Algorithm for Multiple Load Tests

**Figure 11:**
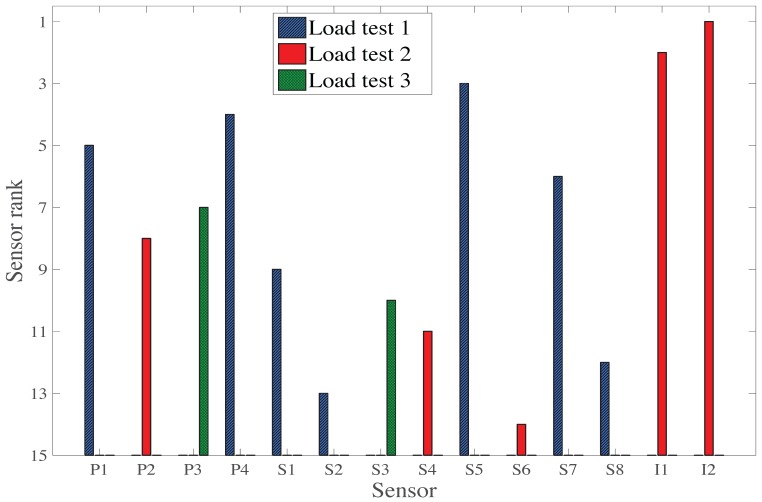
Sensor ranking for the original hierarchical algorithm using three load tests without considering their mutual information.

As mentioned above, this sensor placement algorithm selects a sensor, which is associated to a load test. It does not take into account mutual information between load tests, but can still choose the best load test for each sensor. The sensor ranking of the original hierarchical algorithm for multiple load tests is presented in Figure 11. The selection of sensors is presented as a histogram with the sensor rank in the vertical axis and the sensor identification in the horizontal axis. For each sensor, the load test selected by the sensor placement algorithm is presented as a distinctive bar. As for the single load test consideration, the sensor placement algorithm selects sensors of three different types within the fourth best sensors, confirming that each sensor types provides a unique information.

The two first sensors selected are the inclinometers associated with LT2. The next four sensors selected are strain gauges and deflection targets associated with LT1. The third load test is only considered for the selection of the seventh (P3) and tenth (S3) sensors. In total, five sensors are associated with LT2, seven with LT1, and only two with LT3. As the hierarchical algorithm selects sensors associated with different load tests, it shows that each load test provides unique information. However, LT3 may not provide any substantial additional information as it is only selected at the seventh and tenth sensor.

**Figure 12:**
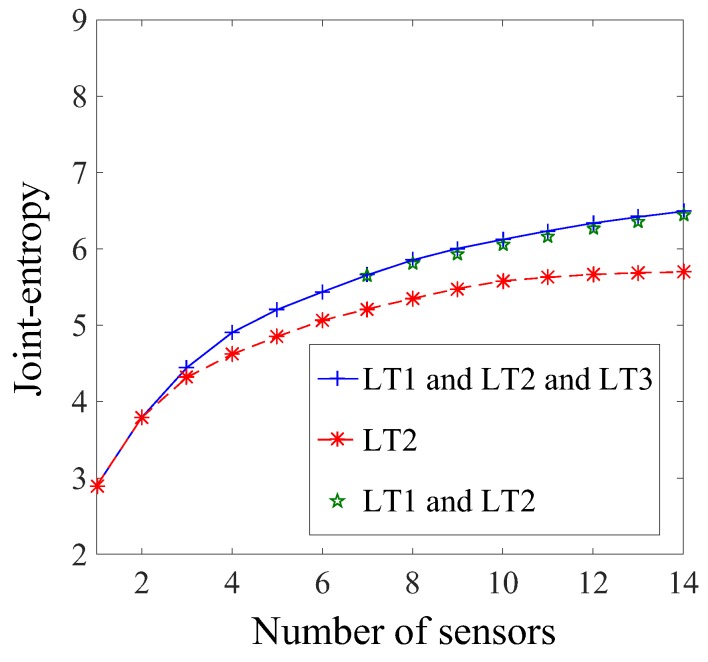
Joint entropy of the sensor configuration as a function of the number of sensors, considering successively the original algorithm for LT2; LT1 and LT2; and all three load tests.

Figure 12 presents a comparison of sensor configurations in terms of joint entropy as a function of the number of sensors using the original hierarchical algorithm for multiple load tests. Three load test configurations are considered: load test 2; load test 1 and load test 2; ad all three load tests. The LT2 was selected for comparison since it provided the largest joint entropy for any number of sensors in the sensor configuration (Figure 10).

When two or three load tests are considered, the joint entropy is larger than considering LT2 alone, showing that each load test provides unique information. However, the difference of joint entropy values between sensor configurations involving three and two load tests is negligible. Using the traditional version of the hierarchical algorithm and considering LT1 and LT2 together provides more information than a single load test. Adding LT3 does not provide significant information compared to LT1 and LT2. Therefore, using this sensor placement strategy, it could be concluded that LT3 should not be carried out.

#### 3.3.2. New Hierarchical Algorithm for Multiple Load Tests

**Figure 13:**
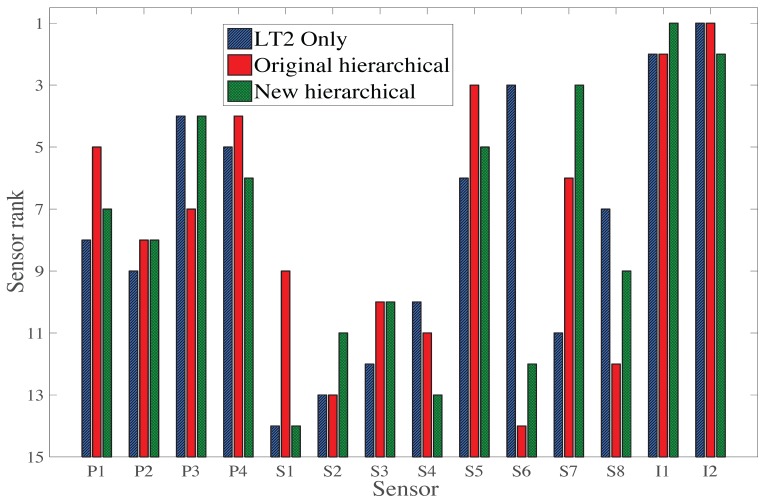
Sensor ranking for the original hierarchical using LT2 independently; the original hierarchical algorithm and the new hierarchical algorithm involving the three load tests.

The sensor ranks of the new hierarchical algorithm using information from the three load tests is compared with the sensor ranks of the original hierarchical algorithm considering only LT2, as well as for multiple load tests, including three load tests (Figure 9 and Figure 11). The sensor rank is presented in Figure 13 on the vertical axis and the sensor identification on the horizontal axis. The new hierarchical algorithm also selects sensors of three different types after adding the fourth best sensor, although the locations of these sensors changed.

The sensor ranking of the new hierarchical algorithm differs already from the first sensor selected, compared with the two versions of the original hierarchical algorithm (Figure 9 and Figure 11). The three sensor placement algorithms select different inclinometer locations: I1 for the new hierarchal algorithm and I2 for the two versions of the original hierarchical algorithm. In the second sensor selection, all sensor placement algorithms select the remaining inclinometers (I2 and I1 respectively). For the third sensor selections, sensor placement algorithms select a strain gauge: S5 for the original hierarchical algorithm for multiple load tests, S6 for the original hierarchical algorithm for single load test, and S7 for the new hierarchical algorithm for multiple load tests. For the fourth sensor selection, a deflection target, P3 for the original hierarchical algorithm for the single load test and the new hierarchical algorithm for multiple load tests, and P4 for the original hierarchical algorithm for multiple load tests are selected. From the fifth sensor onwards, the sensor type selected by sensor placement algorithms differs.

As sensor rankings differ between the original hierarchical algorithm for single load test and the original hierarchical algorithm for multiple load tests, taking into account information from multiple load tests leads to an alternative optimal sensor configuration. Additionally, it shows that sensor rankings differ between the original hierarchical algorithm for multiple load tests and the new hierarchical algorithm for multiple load tests. Taking into account mutual information between load tests leads to an alternative optimal sensor configuration. As optimal sensor configurations differ, a comparison using an information-gain metric, such as the joint entropy metric, is justified.

**Figure 14:**
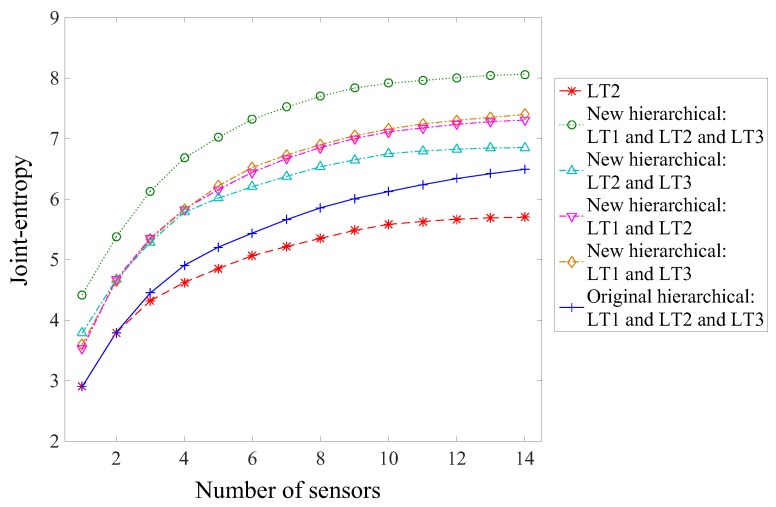
Joint entropy as a function of the number of sensors for various configurations of load tests.

The joint entropy of various load test designs and sensor placement algorithms is presented in Figure 14 as a function of the number of sensors. Results from load test 2 alone using the original hierarchical algorithm are shown for comparison purposes. Various load test designs involving two or three load tests are compared using the new hierarchical algorithm.

Combinations of two or three load tests, using the new hierarchical algorithm, have a significantly larger joint entropy than using only a single load test or a strategy that does not take into account information from all load tests. For any number of sensors, the new hierarchical algorithm provides significantly larger joint entropy values for three load tests than any combinations of two load tests. Therefore, it is shown that the three load tests give unique information. This result is in contradiction with the conclusion from Figure 12, which may lead to a wrong conclusion regarding the utility of a load test.

The three combinations of two load tests present similar results until the sixth sensor is selected. Then, the combination of LT2 and LT3 has a slightly smaller joint entropy, indicating that combinations with LT1 provide more information. Therefore, if only a combination of two load tests has to be considered, LT1 should be included.

### 3.4. Optimal Strategy to Maximize the Information Gain from a Sensor Configuration within a Multiple-Load test Consideration

In the previous sections, the new hierarchical algorithm uses three load tests requiring three measurements for each sensor. However, when the load tests are considered independently in the sensor placement strategy, only one measurement is needed for each sensor. Therefore, to compare these sensor placement strategies in terms of joint entropy, Figure 14 is modified by replacing the number of sensors with the number of measurements (Equation (13)). N_M_ is the number of measurements, N_S_ is the number of sensors, and N_LT_ is the number of load tests used in the sensor placement strategy. The aim is to find the sensor configuration from sensor placement strategies with the largest joint entropy with respect to the number of measurements:

(13) $$N_{M}=N_{S}\times N_{LT}$$

**Figure 15:**
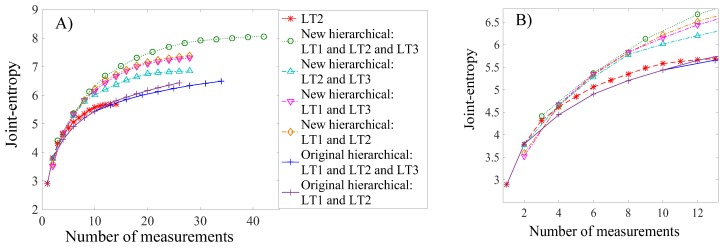
Joint entropy as a function of the number of measurements for various sensor placement methodologies involving various number of load tests (**A**). (**B**) Zoom-in of the figure on the left.

The joint entropy for several sensor placement methodologies involving various load tests is compared with respect to the number of measurements. Results are presented in Figure 15, where, on the right, there is a zoom-in of the region involving a small number of measurements ($N_{M}$ < 13). For each sensor placement methodology, the number of measurements for the same number of sensors differ. The original hierarchical algorithm, considering only LT2, uses one measurement for each sensor added to the configuration, while the new hierarchical algorithm uses three measurements. The sequential process of the original hierarchical algorithm for sensor placement implies the selection of the second and third load tests at different steps of the sensor placement iteration (Figure 11). Therefore, the number of measurements per sensor added to the configuration depends on the step of the sensor-selection process. During the first two sensor selections, the space between markings is equal to one, because only one load test is currently considered. Then, between the third and sixth sensor selections, the space between markings is equal to two, as two load tests are presently considered. This means that two measurements are added per additional sensor to the sensor configuration. Eventually, from the seventh sensor selection, the spacing between markings is equal to three, as three load tests are considered.

The new hierarchical algorithm with the three load tests performs the best for any number of measurements, whereas for two of the three load tests the algorithm performs better than the other sensor placement strategies for any number of measurements. All sensor placement strategies present similar results for a small number of measurements (N_M_ < 4); adding a new sensor or a new load test provides almost the same amount of information. However, for a larger number of measurements, a strategy using information from multiple load tests and various sensor types performs better, compared with adding new sensors to the configuration. Thus, a strategy accounting for various sensor types and load test configurations performs best in terms of information gain.

## 4. Discussion

In this section, a comparison of the sensor ranking using the various sensor placement strategies in this study is presented and the limitations are discussed. A sensor placement strategy based on the joint entropy is able to identify the sensor configuration which will be the most beneficial to identify the parameters of a system, which is, in this case, a bridge. Outcomes of structural identification, such as reserve capacity estimation, are highly dependent on the quality of field measurements. The use of a sensor placement methodology may, thus, ensure that the maximum of information will be gained during load testing. Additionally, an advantage of both the original and the new hierarchical algorithms is the monotonic and bounded properties of the joint entropy objective function. Thus, a stop criterion, for instance, an increase of joint entropy between two sensor configurations smaller than a threshold, could be introduced to identify an optimal number of sensors. By decreasing the number of sensors, the cost of monitoring could often be reduced, guaranteeing that the information gain is not compromised. 

Table 3 displays a summary of the sensor ranking from the various sensor placement strategies proposed in this study. Except for the case of the first load test (LT1), all sensor placement strategies select sensor types in the same order: two inclinometers, then a strain gauge and a deflection target. However, the selection of sensor locations differs, indicating that this aspect is very sensitive to the choice of sensor placement strategy. If multiple static load tests are planned, a sensor placement strategy, which does not take into account information from multiple load tests, may lead to a sub-optimal sensor configuration. Additionally, the original hierarchical algorithm suggests that LT3 does not provide additional information compared with LT1 and LT2 (Figure 12), while the new hierarchical algorithm suggests that LT3 provides unique information (Figure 14). This contradiction shows that the uniqueness of information is difficult to estimate when the mutual information among multiple load tests is not evaluated, and this may lead to the wrong decision on, for example, whether or not to add a new test to load testing plans. 

Both the original and the new hierarchical algorithms ranks highly all sensor types, even if individual information entropy values are small (Figure 8). This means that each sensor type provides unique information. For a given number of measurements, placing multiple sensor types, associated with several load tests, is, thus, the best strategy to maximize information gain during a field measurement campaign.

The following limitations of the work are recognized: The greedy algorithm used in the three sensor placement strategies does not necessarily lead to a global optimum. Moreover, the sampling technique and the estimation of modelling uncertainties at sensor locations influence the results. Finally, only fourteen locations were investigated, due to practical considerations which restricted the sensor installation to some parts of the bridge. Research is underway to assess the impact of these aspects on the results.

Another important restriction of any model-based sensor placement methodology is that the success of the study depends on the quality of the numerical model used to obtain predictions. Therefore, it is primordial to build a reliable model to get trustful predictions at possible sensor locations. Model assumptions should be verified during visual inspection, before load testing the bridge. Additionally, test configurations, such as possible load tests and available sensor types and numbers, should be defined sufficiently in advance of computing the model instance predictions and running the sensor placement algorithm to obtain the optimal sensor configuration.

## 5. Conclusions

A rational sensor placement methodology can increase the performance of the structural identification methodology by enhancing the model instance discrimination. Specific conclusions are as follows:

- Optimal sensor configuration strategies should include static-load test characteristics.
- When there are multiple load tests, a sensor placement strategy should take this into account for optimal sensor configuration. Failure to do so may lead to the wrong conclusions in terms of information gain, for example, when deciding whether or not to add a new test to the load testing plans.
- A strategy including the combination sensor types and load test configurations increases the information gain compared to strategies that do not account for such interaction.

Future work will focus on the combination of static and dynamic load tests for sensor configuration, as strain-gauge sensors were used in both tests. Additionally, a multi-criteria decision-making approach will be developed to include cost, information gain criteria, and sensor installation costs in the determination of optimal sensor configurations for a range of case studies.

## Figures and Tables

**Table 1:** Primary parameters considered and their initial intervals.

E_con_ (GPa)	E_pre_ (GPa)	E_bar_ (GPa)	K_rot_ log(Nmm/rad)	K_lon_ log(N/mm)
20–35	25–50	3–40	9–13	8–11

**Table 2:** Modelling and measurement uncertainties.

Uncertainty Source	Displacements—(P)		Rotations—(I)		Strains—(S)	
	Min	Max	Min	Max	Min	Max
Model simplifications (%)	−5	13	−5	13	−5	13
Mesh refinement (%)	−1	1	−1	1	−1	1
Spatial variability (%)	-	-	-	-	−5	5
Additional uncertainty (%)	−1	1	−1	1	−1	1
Sensor precision	−0.05 mm	0.05 mm	−1 μrad	1 μrad	−2 με	2 με
Repeatability	−0.15 mm	0.15 mm	−4 μrad	4 μrad	−4 με	4 με
Sensor orientation (%)	-	-	-	-	0	6
Sensor installation (%)	-	-	−5	5	0	5

**Table 3:** Summary of sensor ranking from the various sensor placement strategies.

Sensor placement Strategy	Sensor Ranking													
	1	2	3	4	5	6	7	8	9	10	11	12	13	14
Load test 1 only—Hierarchical algorithm	I2	P1	S5	I1	P4	S7	S1	P2	S2	S8	P3	S6	S4	S3
Load test 2 only—Hierarchical algorithm	I2	I1	S6	P3	P4	S5	S8	P1	P2	S4	S7	S3	S2	S1
Load test 3 only—Hierarchical algorithm	I1	I2	S4	P3	S8	P4	P1	S7	P2	S5	S3	S2	S6	S1
LT1 & LT2 & LT3—Original hierarchical	I2	I1	S5	P4	P1	S7	P3	P2	S1	S3	S4	S8	S2	S6
LT1 & LT2—Original hierarchical	I2	I1	S5	P4	S7	P1	P3	S1	S2	S4	P2	S6	S3	S8
LT1 & LT2 & LT3—New hierarchical	I1	I2	S7	P3	S5	P4	P1	P2	S8	S3	S2	S6	S4	S1
LT1 & LT2—New hierarchical	I1	I2	S7	P4	S5	P2	P1	S8	P3	S4	S2	S6	S1	S3
LT1 & LT3—New hierarchical	I1	I2	S7	P3	S5	P4	P1	S8	P2	S4	S2	S6	S1	S3
LT2 & LT3—New hierarchical	I1	I2	S4	P3	P4	P1	P2	S8	S5	S6	S3	S7	S2	S1
